# Association of Pediatric Buccal Epigenetic Age Acceleration With Adverse Neonatal Brain Growth and Neurodevelopmental Outcomes Among Children Born Very Preterm With a Neonatal Infection

**DOI:** 10.1001/jamanetworkopen.2022.39796

**Published:** 2022-11-02

**Authors:** Noha Gomaa, Chaini Konwar, Nicole Gladish, Stephanie H. Au-Young, Ting Guo, Min Sheng, Sarah M. Merrill, Edmond Kelly, Vann Chau, Helen M. Branson, Linh G. Ly, Emma G. Duerden, Ruth E. Grunau, Michael S. Kobor, Steven P. Miller

**Affiliations:** 1 Schulich School of Medicine & Dentistry, Western University, London, Ontario, Canada; 2 Neuroscience and Mental Health Program, SickKids Research Institute, Hospital for Sick Children, Toronto, Ontario, Canada; 3 Department of Medical Genetics, University of British Columbia, Vancouver, British Columbia, Canada; 4 Division of Neonatology, Mount Sinai Hospital, Toronto, Ontario, Canada; 5 Department of Pediatrics, University of Toronto, Toronto, Ontario, Canada; 6 Department of Diagnostic Imaging, Hospital for Sick Children, Toronto, Ontario, Canada; 7 Department of Medical Imaging, University of Toronto, Toronto, Ontario, Canada; 8 Division of Neonatology, Hospital for Sick Children, Toronto, Ontario, Canada; 9 Faculty of Education, Western University, London, Ontario, Canada; 10 Division of Neonatology, BC Children’s Hospital, Vancouver, British Columbia, Canada; 11 Department of Pediatrics, University of British Columbia, Vancouver, British Columbia, Canada; 12 British Columbia Children’s Hospital Research Institute, Vancouver, British Columbia, Canada

## Abstract

**Importance:**

Very preterm neonates (24-32 weeks’ gestation) remain at a higher risk of morbidity and neurodevelopmental adversity throughout their lifespan. Because the extent of prematurity alone does not fully explain the risk of adverse neonatal brain growth or neurodevelopmental outcomes, there is a need for neonatal biomarkers to help estimate these risks in this population.

**Objectives:**

To characterize the pediatric buccal epigenetic (PedBE) clock—a recently developed tool to measure biological aging—among very preterm neonates and to assess its association with the extent of prematurity, neonatal comorbidities, neonatal brain growth, and neurodevelopmental outcomes at 18 months of age.

**Design, Setting, and Participants:**

This prospective cohort study was conducted in 2 neonatal intensive care units of 2 hospitals in Toronto, Ontario, Canada. A total of 35 very preterm neonates (24-32 weeks’ gestation) were recruited in 2017 and 2018, and neuroimaging was performed and buccal swab samples were acquired at 2 time points: the first in early life (median postmenstrual age, 32.9 weeks [IQR, 32.0-35.0 weeks]) and the second at term-equivalent age (TEA) at a median postmenstrual age of 43.0 weeks (IQR, 41.0-46.0 weeks). Follow-ups for neurodevelopmental assessments were completed in 2019 and 2020. All neonates in this cohort had at least 1 infection because they were originally enrolled to assess the association of neonatal infection with neurodevelopment. Neonates with congenital malformations, genetic syndromes, or congenital TORCH (toxoplasmosis, rubella, cytomegalovirus, herpes and other agents) infection were excluded.

**Exposures:**

The extent of prematurity was measured by gestational age at birth and PedBE age difference. PedBE age was computed using DNA methylation obtained from 94 age-informative CpG (cytosine-phosphate-guanosine) sites. PedBE age difference (weeks) was calculated by subtracting PedBE age at each time point from the corresponding postmenstrual age.

**Main Outcomes and Measures:**

Total cerebral volumes and cerebral growth during the neonatal intensive care unit period were obtained from magnetic resonance imaging scans at 2 time points: approximately the first 2 weeks of life and at TEA. Bayley Scales of Infant and Toddler Development, Third Edition, were used to assess neurodevelopmental outcomes at 18 months.

**Results:**

Among 35 very preterm neonates (21 boys [60.0%]; median gestational age, 27.0 weeks [IQR, 25.9-29.9 weeks]; 23 [65.7%] born extremely preterm [<28 weeks’ gestation]), extremely preterm neonates had an accelerated PedBE age compared with neonates born at a later gestational age (β = 9.0; 95% CI, 2.7-15.3; *P* = .01). An accelerated PedBE age was also associated with smaller cerebral volumes (β = –5356.8; 95% CI, −6899.3 to −2961.7; *P* = .01) and slower cerebral growth (β = –2651.5; 95% CI, −5301.2 to −1164.1; *P* = .04); these associations remained significant after adjusting for clinical neonatal factors. These findings were significant at TEA but not earlier in life. Similarly, an accelerated PedBE age at TEA was associated with lower cognitive (β = –0.4; 95% CI, −0.8 to −0.03; *P* = .04) and language (β = –0.6; 95% CI, −1.1 to −0.06; *P* = .02) scores at 18 months.

**Conclusions and Relevance:**

This cohort study of very preterm neonates suggests that biological aging may be associated with impaired brain growth and neurodevelopmental outcomes. The associations between epigenetic aging and adverse neonatal brain health warrant further attention.

## Key Points

**Question:**

Is epigenetic aging associated with neonatal brain growth and neurodevelopmental outcomes among very preterm neonates?

**Findings:**

In this cohort study of 35 neonates, accelerated epigenetic aging—measured by the pediatric buccal epigenetic clock—was associated with smaller brain volumes at term-equivalent age and slower brain growth during the neonatal period, as well as worse neurodevelopmental outcomes at 18 months of age.

**Meaning:**

This study suggests that biological epigenetic aging among very preterm neonates may be an indicator of brain growth and neurodevelopmental outcomes, and its application requires further investigation.

## Teaser

This cohort study characterizes the pediatric buccal epigenetic clock among very preterm neonates and assesses its association with the extent of prematurity, neonatal comorbidities, neonatal brain growth, and neurodevelopmental outcomes at 18 months of age.

## Introduction

Very preterm birth constitutes a risk for neonatal comorbidities (eg, infections, chronic lung disease, and brain injury), and has been strongly associated with structural and functional changes in the brain, including altered growth, metabolism, and connectivity, which are pivotal indicators of neurodevelopmental trajectories.^1,2^ Generally, the extent of prematurity is used as an indicator of neurodevelopment for children born very preterm, including motor, language, and cognitive abilities.^3,4,5,6^ However, studies have suggested that the extent of prematurity alone does not fully explain alterations in the neonatal brain.^7^ Similarly, illnesses related to preterm birth, such as bronchopulmonary dysplasia, and brain injuries, such as intraventricular hemorrhage or white matter injury, although important, only partly explain brain dysmaturation from early in life to term-equivalent age (TEA).^7^ There is, therefore, a need for instruments that extend beyond the existing clinical factors and medical assessments in the neonatal intensive care unit (NICU) to help understand this variation among children born very preterm. Epigenetic-based biomarkers have been postulated to provide insights into neonatal and child development and to provide an additional dimension to investigating the risk to neurodevelopment.^8,9,10,11^

The most commonly investigated epigenetic marker in humans is DNA methylation, which is the chemical addition of a methyl group to the cytosine residue, typically at cytosine-phosphate-guanosine (CpG) dinucleotides. Epigenome-wide association studies of preterm infants have found an association between DNA methylation changes at specific CpG sites and neural function and signaling, such as within the neural-relevant genes *SLC7A5* (human; NCBI NC_000016.10), *SLC1A2* (human; NCBI NC_000011.10), *APOL1* (human; NCBI NC_000022.11), *TMEM266* (human; NCBI NC_000015.10), and *TENM2* (human; NCBI NC_000005.10).^12,13^ These have been associated with changes in white matter tract tissue integrity and shape and NICU comorbidities, including bronchopulmonary dysplasia, severe brain injury, neonatal infections, and severe retinopathy of prematurity (ROP).^14^ Further studies of children born preterm have also revealed epigenome-wide associations with gestational age (GA), birth weight, and brain dysmaturation,^15,16,17^ in which variation in DNA methylation explained the association between preterm birth and the dysconnectivity of developing brain networks that characterize the atypical development of the preterm brain.^17^ Another approach to harness DNA methylation in association with development and health is the epigenetic clocks, which are a class of biological age estimators that use the level of DNA methylation at a set of computationally determined age-related CpG sites to estimate the biological epigenetic age.^18^ The principal notion is to assess whether there are differences between the estimated epigenetic age and the actual chronological age of an individual.^18^ Such differences would then indicate individual variation in biological aging that can possibly be associated with experiences and environments, on the one hand, or health outcomes, on the other. Clinical and epidemiological studies have found that accelerated epigenetic age, in which computationally estimated epigenetic age is “older” than chronological age, can be used to estimate mortality risk and associations with several health problems among adults (eg, frailty, cancer, cardiovascular disease, obesity, asthma, neurologic diseases, and structural brain changes, such as cortical thinning).^19,20,21,22,23,24^ Limited studies have also used these adult epigenetic clocks for children, reporting an acceleration of epigenetic aging in association with neurodevelopmental conditions and affective disorders.^25,26^ The use of adult epigenetic clocks in the pediatric population, however, represents a methodological challenge because of the large differences in DNA methylation patterns between children and adults, which limits the validity of the adult epigenetic clock in studying child exposures and outcomes.^27^ To address these challenges, the pediatric buccal epigenetic (PedBE) clock was recently developed from noninvasive buccal epithelial swab samples. The PedBE clock is based on DNA methylation at 94 age-informative CpG sites, which accurately estimate chronological age among normative, healthy, and full-term children.^28^ Combining these DNA methylation values as a polyepigenetic measure of cellular aging has been found to be more associated with aging than each age-informing CpG individually.^18,28^ In a recent independent evaluation of epigenetic clocks, the PedBE clock showed the highest accuracy with chronological age in pediatric buccal samples.^29^ Initial studies using the PedBE clock have shown accelerated epigenetic aging among children with autistic spectrum disorder^28^ and internalizing disorders.^30^ An accelerated PedBE age has also been recently suggested as a potential prognostic biomarker for pediatric brain tumors.^29^ However, testing the extent to which the PedBE clock can report on brain growth and development during the neonatal period, particularly in the preterm population, has not been assessed, to our knowledge.

In this study, we sought to characterize the PedBE clock in a cohort of very preterm neonates. We hypothesized that the difference between the PedBE clock–estimated epigenetic age and chronological age—referred to hereafter as *the difference in PedBE age*—will be associated with adverse neonatal brain growth and neurodevelopmental outcomes at 18 months of age, corrected for GA. Our specific objectives were to (1) evaluate whether the extent of prematurity and neonatal comorbidities in early life and at TEA are associated with an accelerated PedBE age; (2) assess whether an accelerated PedBE age is associated with neonatal total cerebral volume (TCV) and TCV growth, independent of clinical comorbidities, and whether this association differs between early in life and TEA; and (3) evaluate whether an accelerated PedBE age is associated with neurodevelopmental outcomes at 18 months’ corrected age.

## Methods

### Study Design, Setting, and Participants

For this prospective cohort study, we used the Strengthening the Reporting of Observational Studies in Epidemiology (STROBE) reporting guidelines.^31^ Neonates born very preterm (24-32 weeks’ gestation) admitted to the NICUs at the Hospital for Sick Children and Mount Sinai Hospital, Toronto, Ontario, Canada, were enrolled in 2017 and 2018. Follow-up for neurodevelopmental assessments was completed in 2019 and 2020. Both hospitals are connected by a tunnel so that neonates from each NICU are studied using the same magnetic resonance imaging (MRI) scanner. Because this cohort was originally enrolled to assess the association of neonatal infection with neurodevelopment, all neonates studied had at least 1 infection: histologic chorioamnionitis, clinical sepsis, culture-positive sepsis, or other culture-positive infection.^32^ Neonates with congenital malformations or evidence of a genetic syndrome or those with a congenital TORCH (toxoplasmosis, rubella, cytomegalovirus, herpes and other agents) infection were not enrolled. Research ethics boards at both hospitals approved the study, and written informed consent was obtained from parents or legal caregivers. Demographic data and clinical variables were systematically collected by neonatal research nurses from electronic health records in each NICU on a daily basis and recorded in our central laboratory database in RedCap.^33^ Consenting parents provided sociodemographic information, including self-reported racial and ethnic background, language spoken at home, and level of maternal and paternal education.

### Extent of Prematurity

Based on GA, we split the cohort into 2 groups: (1) extremely preterm (<28 weeks’ gestation) and (2) very preterm (28-32 weeks’ gestation). This cutoff is based on the clinical literature suggesting that neonates born before 28 weeks’ gestation have different growth patterns and health outcomes than neonates born at a later GA.^34,35,36^

### Magnetic Resonance Imaging

Neonates underwent MRI without sedation in a neonatal MRI transport incubator (Sree Medical Systems) on the same Siemens 3 T Tim Trio MRI scanner at the Hospital for Sick Children, Toronto. A single-channel neonatal head coil was used (Sree Medical Systems) (eMethods in the Supplement).

### Volumetric Measures of the Brain

Anatomical images were reviewed by a pediatric neuroradiologist (H.M.B.) and pediatric neurologists (V.C. and S.P.M.). Total cerebral volumes were obtained on each participant’s T1-weighted MRI by manual segmentation using the 3-dimensional visualization software Display^37^ as previously described.^38,39^ Total cerebral volume growth was calculated as (TCV at MRI scan 2 − TCV at MRI scan 1)/(postmenstrual age [PMA] at MRI scan 2 − PMA at MRI scan 1).

### Neurodevelopmental Outcomes at 18 Months

Neurodevelopmental abilities were assessed using the Bayley Scales of Infant and Toddler Development, Third Edition (Bayley-III).^40^ We collected cognitive, language, and motor composite scores in follow-up appointments at 18 months corrected for GA.

### PedBE Clock

#### DNA Extraction and DNA Methylation Quantification

Buccal epithelial swab samples were obtained from very preterm neonates at the same time as their MRI scans (ie, MRI scan 1 within the first 2 weeks of life and then again at MRI scan 2 at TEA). We extracted DNA from the buccal swab samples using the Isohelix Buccal-Prep Plus DNA isolation kits, followed by bisulfite conversion using the Zymo EZ NA methylation kit. To obtain the DNA methylation measurements at the PedBE clock sites, we used the Illumina Infinium Human MethylationEPIC BeadChip platform (850K Array).^41^ Further details on sample preprocessing are described in the eMethods in the Supplement.

#### Epigenetic Age Estimation

After sample preprocessing and correcting for background fluorescence and dye bias,^42^ we calculated PedBE age using the online script publicly available on the GitHub repository.^43^ PedBE age difference was calculated as the difference between the estimated epigenetic age and PMA (weeks) at each time point (MRI scan 1 and MRI scan 2), thereby providing 2 difference in PedBE age values for each neonate. Details of genetic ancestry assessment and cell-type estimation are described in the eMethods and eFigure 1 in the Supplement.

### Statistical Analysis

We split our sample into 2 groups based on GA: extremely preterm neonates (born at <28 weeks’ gestation) and very preterm neonates (born at 28-32 weeks’ gestation), and we compared clinical neonatal factors, TCV, TCV growth, and neurodevelopmental outcomes between these 2 categories using the Kruskal-Wallis test for continuous variables and the Fisher exact test for categorical variables. We used the Spearman rank correlation coefficient to assess the correlation of the PedBE age with GA and PMA, and whether these correlations differed by neonatal sex. Next, we constructed generalized linear models to assess the univariate association between the extent of prematurity and the PedBE age difference in weeks as the outcome variable, at each of the 2 neonatal time points (MRI scan 1 and MRI scan 2). We then used generalized linear regression models to assess the association of PedBE age difference with TCV and TCV growth as the outcome variables at each of the neonatal time points, and then we adjusted for the clinical comorbidities that have been previously associated with impaired brain growth, including bronchopulmonary dysplasia, patent ductus arteriosus, infection, moderate to severe brain injury, severe ROP, in separate models, controlling for 1 covariate at a time. Descriptions of the outcome variables and case definitions of comorbid conditions are available in the eMethods in the Supplement. All models were controlled for GA. Regression coefficients, 95% CIs, and nominal *P* values for each time point were then compared. Finally, we assessed the association of PedBE age difference with neurodevelopmental outcomes at 18 months of age using generalized linear regressions. All DNA methylation preprocessing, epigenetic age estimation, and ancestry and cell-type assessment were performed in R, version 3.6 (R Group for Statistical Computing). Statistical analyses were conducted in Stata, version 16.1 (StataCorp LLC). All *P* values were from 2-sided tests and results were deemed statistically significant at *P* < .05.

## Results

### Cohort Characteristics

This cohort study included 35 very preterm neonates with a median GA of 27.0 weeks (IQR, 25.9-29.9 weeks). Of these, 21 were male (60.0%), 23 (65.7%) were born at less than 28 weeks’ gestation, and 8 (22.9%) had 3 or more prenatal and/or postnatal infections (Table 1).^44^ Neonates underwent MRI scans and had buccal swab samples obtained at 2 neonatal time points: early in life (PMA, 32.9 weeks [IQR 32.0-35.0 weeks]) and at TEA (PMA, 43.0 weeks [IQR, 41.0-46.0 weeks]). No significant differences were evident in any of the examined clinical characteristics by biological sex. Extremely preterm neonates did not exhibit significant differences in comorbidities compared with those born at a later GA, except for a greater incidence of patent ductus arteriosus (19 of 23 [82.6%] vs 2 of 12 [16.7%]; *P* < .001). At 18 months’ follow-up, median Bayley-III scores were below the age-expected norms for children born extremely preterm and those born very preterm, and 4 infants (11.4%) received a diagnosis of cerebral palsy.

**Table 1.** Cohort Characteristics According to Extent of Prematurity

Characteristic	Neonates, No. (%) (N = 35)		*P* value
	Born at ≥28 wk gestation (n = 12)	Born at <28 wk gestation (n = 23)	
GA, median (IQR), wk	28.8 (28.1-30.1)	25.7 (24.7-27)	NA
Sex			
Female	7 (58.3)	7 (30.4)	
Male	5 (41.7)	16 (69.6)	.34
Maternal level of education, ≥postsecondary education	5 (41.7)	14 (60.9)	.13
Maternal smoking, yes	3 (25.0)	8 (34.8)	.87
PDA	2 (16.7)	19 (82.6)	<.001
BPD	2 (16.7)	4 (17.4)	.56
Severe ROP (≥stage III)	1 (8.3)	6 (26.1)	.35
≥3 Infections	1 (8.3)	7 (30.4)	.09
Moderate to severe brain injury^a^	6 (50.0)	15 (65.2)	.27
Total cerebral volume, median (IQR), mm^3^			
At MRI scan 1	190 000 (180 000-220 000)	180 000 (160 000-200 000)	.10
At MRI scan 2	410 000 (340 000-460 000)	350 000 (280 000-410 000)	.07
Bayley-III score, median (IQR)			
Cognitive scale at 18 mo	95.0 (85.0-105.0)	90.0 (85.0-100.0)	.91
Language scale at 18 mo	87.5 (79.0-100.0)	89.0 (77.0-97.0)	.77
Motor scale at 18 mo	97.0 (76.0-100.0)	94.0 (85.0-100.0)	.96

Abbreviations: Bayley-III, Bayley Scales of Infant and Toddler Development, Third Edition; BPD, bronchopulmonary dysplasia; GA, gestational age; MRI, magnetic resonance imaging; NA, not applicable; PDA, patent ductus arteriosus (defined as echocardiographic evidence of a PDA shunt); ROP, retinopathy of prematurity.

^a^ Moderate to severe brain injury: any severity of punctate white matter injury^44^ or intraventricular hemorrhage of grade II or higher.

### Characterization of PedBE Age in Study Cohort

Given the novelty of the PedBE clock, we first characterized it in this clinical population (n = 35). At both time points, early in life and at TEA, PedBE age showed an inverse correlation with GA with no biological sex-specific differences. A positive correlation was observed between PedBE age and PMA early in life (*r* = 0.36; *P* = .01) and at TEA (*r* = 0.68; *P* < .001) (eFigure 2 in the Supplement).

### Extent of Prematurity and NICU Comorbid Conditions Associated With an Accelerated PedBE Age

**Figure 1.**
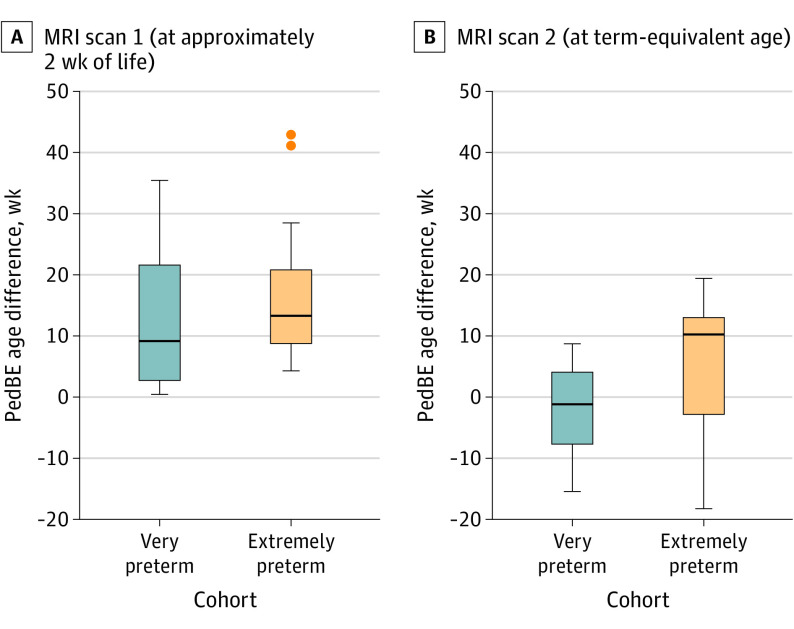
Pediatric Buccal Epigenetic (PedBE) Age Difference in Association With Extent of Prematurity Extremely preterm neonates (24.7-27 weeks’ gestation) showed an accelerated PedBE age compared with neonates born at a later gestational age (28.1-30.1 weeks’ gestation) at term-equivalent age (*P* = .01). No significant PedBE age difference between extremely preterm and very preterm neonates was observed within the first 2 weeks of life. The boxes indicate the difference in PedBE age distribution; the lines indicate the range of difference in PedBE age with upper and lower limit values; and the dots indicate distribution across range (outliers are past upper or lower limit). MRI indicates magnetic resonance imaging.

Generalized linear models to assess the univariate association of the extent of prematurity with PedBE age difference (weeks) showed PedBE age to be significantly accelerated among extremely preterm neonates compared with their counterparts born at a later GA at TEA (β = 9.0; 95% CI, 2.7-15.3; *P* = .01) (Table 2). However, this association was not significant at the time of the initial MRI scan conducted within the first 2 weeks of life (Figure 1). An accelerated PedBE age at TEA was also associated with patent ductus arteriosus (β = 7.3; 95% CI, 1.1-13.4; *P* = .01) and severe ROP (β = 9.3; 95% CI, 1.1-17.6; *P* = .02) but not with other clinical comorbid conditions (Table 2).

**Table 2.** Results of Univariate Generalized Linear Models Showing Associations Between an Accelerated PedBE Age and the Extent of Prematurity and Neonatal Clinical Factors at Term-Equivalent Age

Neonatal clinical factor	PedBE age difference, wk	
	β (95% CI)^a^	*P* value
Extreme prematurity (<28 wk gestation)	9.0 (2.7 to 15.3)	.01
Maternal educational level	−1.4 (−8.4 to 5.5)	.69
Maternal smoking	−0.4 (−3.5 to 2.5)	.75
PDA	7.3 (1.1 to 13.4)	.01
BPD	5.1 (3.5 to 11.1)	.12
Severe ROP (≥stage III)	9.3 (1.1 to 17.6)	.02
≥3 Infections	−1.6 (−9.5 to 6.2)	.68
Moderate to severe brain injury	−0.9 (−7.5 to 5.6)	.78

Abbreviations: BPD, bronchopulmonary dysplasia; PDA, patent ductus arteriosus; PedBE, pediatric buccal epigenetic clock; ROP, retinopathy of prematurity.

^a^ Magnitude of change in PedBE age difference in weeks with every unit change in the listed neonatal clinical factors.

### Association of Accelerated PedBE Age With Neonatal TCVs and TCV Growth

**Figure 2.**
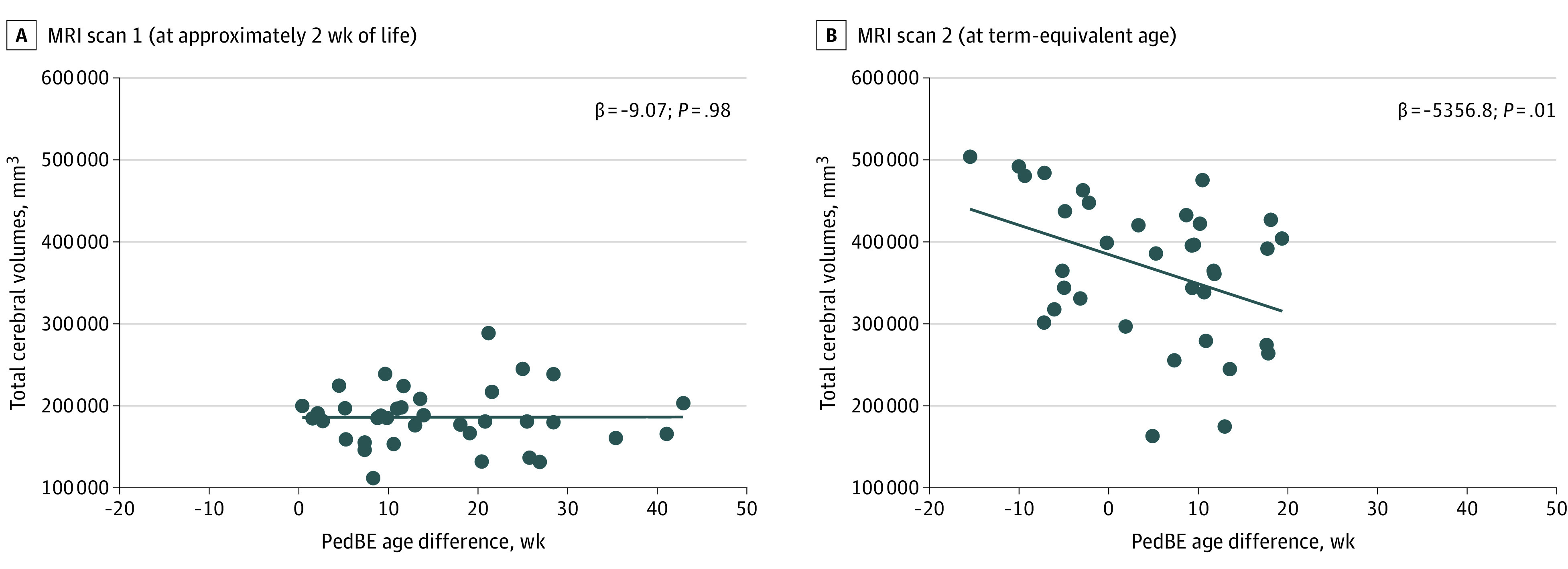
Pediatric Buccal Epigenetic (PedBE) Age Difference in Association With Brain Growth PedBE age was significantly accelerated in association with smaller total cerebral volumes at term-equivalent age (magnetic resonance imaging [MRI] scan 2) but not within the first 2 weeks of life (MRI scan 1). In each panel, the dots indicate the values for total cerebral volumes regressed on difference in PedBE age, and the line indicates the line of best fit.

We used a generalized linear model to quantify the association of PedBE age difference (weeks) with TCV and TCV growth (millimeters cubed), accounting for GA and a priori determined comorbidities. In the crude model, we found an accelerated PedBE age to be associated with smaller TCV (β = −5356.8; 95% CI, −6899.3 to −2961.7; *P* = .01) and slower TCV growth (β = –2651.5; 95% CI, −5301.2 to −1164.1; *P* = .04) at TEA (Figure 2). These associations remained significant after adjusting for GA and clinical factors that have been previously associated with impaired TCV (Table 3). The association between TCV growth and PedBE age difference was attenuated by adjusting for patent ductus arteriosus and severe ROP.

**Table 3.** Results of Generalized Linear Models Showing Association of PedBE Age Difference With TCV and TCV Growth at Term-Equivalent Age

Independent variable	TCV, mm^3^		TCV growth, mm^3^	
	β (95% CI)^a^	*P* value	β (95% CI)^a^	*P* value
Model 1				
PedBE age dfference^b^	−4930.6 (−6369.7 to −3491.5)	.01	−3351.5 (−6549.0 to −153.7)	.04
Model 2				
PedBE age difference				
A	−4353.7 (−8019.8 to −687.6)	.04	−3257.4 (−6195.6 to 80.8)	.05
B	−3282.0 (−6554.5 to −3571.1)	.04	−3257.4 (−6595.7 to −808.8)	.03
C	−5073.5 (−6564.4 to −3582.6)	.03	−48382.7 (−11623.7 to 24681.03)	.19
D	−5051.9 (−6463.9 to −3640.0)	.003	−3893.6 (−7039.9 to −747.4)	.01
E	−4723.8 (−6254.2 to −3193.4)	.02	−3396.9 (−6626.7 to −167.2)	.03

Abbreviations: PedBE, pediatric buccal epigenetic clock; TCV, total cerebral volume.

^a^ Magnitude of change in TCV and TCV growth per 1 week of PedBE age difference (millimeters cubed per week).

^b^ Model 1 is adjusted for gestational age. Model 2 (A-E) further adjusts for the clinical factors involved in TCV in separate models (n = 35): A, adjusted for GA and patent ductus arteriosus; B, adjusted for gestational age and bronchopulmonary dysplasia; C, adjusted for gestational age and severe retinopathy of prematurity; D, adjusted for gestational age and number of infections; and E, adjusted for gestational age and moderate to severe brain injury.

### Association of Accelerated PedBE Age With Adverse Neurodevelopmental Outcomes at 18 Months

In multivariable linear regression models, an accelerated PedBE age at TEA was associated with lower Bayley-III cognitive and language scores at 18 months of age (cognitive score: β = –0.4; 95% CI, −0.8 to −0.03; *P* = .04; language score: β = –0.6; 95% CI, −1.1 to −0.06; *P* = .03) but not with motor scores (β = 0.005; 95% CI, −0.5 to 0.56; *P* = .98). Adjusting for severe ROP attenuated the association between an accelerated PedBE age and cognitive (β = –0.2; 95% CI, −0.6 to 0.2; *P* = .37) and language (β = –0.4; 95% CI, −1.0 to 0.09; *P* = .09) scores.

## Discussion

In a cohort of deeply phenotyped very preterm neonates (24-32 weeks’ gestation), we assessed the PedBE clock in association with brain growth and neurodevelopmental outcomes. Neuroimaging studies have previously provided valuable insights into the determinants of brain growth and neurodevelopmental outcomes in this population.^1,45,46,47,48^ Here, we found extremely preterm neonates (<28 weeks’ gestation) to have an accelerated PedBE age at TEA compared with very preterm neonates (28-32 weeks’ gestation). We also found an association between accelerated PedBE age and smaller neonatal brain volumes and slower brain growth at TEA, as well as adverse cognitive and language Bayley-III outcomes at 18 months’ follow-up.

Extremely preterm neonates are well known to be at a higher risk for neonatal comorbidities and adverse neurodevelopmental outcomes along the lifespan compared with those born at a later GA.^4,35,38,47,49,50^ The stress of preterm birth and related NICU exposures (eg, pain and medications) may further accentuate these risks.^51,52^ Our findings showing extremely preterm neonates to have an accelerated PedBE age suggest that the extent of prematurity may be a determinant of biological aging. These observations are further corroborated by the acceleration of PedBE age in association with NICU comorbid conditions (severe ROP and bronchopulmonary dysplasia), which are consequences of extreme prematurity known for their association with impaired brain health and development.^53,54^ Another neonatal factor that might also have contributed to the acceleration of PedBE age among extremely preterm born neonates is NICU nutrient intake—which depends on GA^55^ and which has been previously linked to epigenetic changes responsible for growth patterns in preterm infants.^55^

Epigenome-wide association studies of preterm neonates have found associations of DNA methylation patterns with altered neural function and signaling, alterations in white matter tract integrity, and NICU comorbidities, suggesting a possible contribution of DNA methylation variation to brain health and neurodevelopment among preterm infants.^15,16,17^ Meanwhile, the advent of pediatric epigenetic clocks, such as the PedBE clock, has facilitated an enhanced understanding of brain disorders as well as behavioral and developmental conditions in the context of biological aging in children.^28,30^ We extend these findings to the neurodevelopment of very preterm neonates by showing associations of the accelerated PedBE age with smaller neonatal brain volumes, slower neonatal brain growth, and adverse cognitive and language skills in early childhood. Although GA is consistently and conventionally used as the fundamental indicator of neonatal development, it may not be the optimal indicator for brain health in the NICU and after discharge, especially for extremely preterm neonates.^7,56^ Although the clinical application of the PedBE clock is challenged by the accessibility of the DNA methylome, subsequent microarray measurements of PedBE clock sites, and the associated time and cost, our results suggest its potential as a complementary tool to the existing clinical methods that can help in understanding brain growth among neonates in the NICU. Further investigations that address the value of the PedBE clock for neonatal brain growth and neurodevelopmental outcomes at follow-up will be essential in advancing the potential clinical application of PedBE.

Our findings suggest that the timing of assessing epigenetic aging in the NICU may be critical, where a significant association between an accelerated PedBE age and brain growth was observed at TEA but not within the first weeks of life. A growing body of research suggests that the timing of exposure may be key in determining the magnitude of DNA methylation change associated with health outcomes.^57,58,59^ In addition, DNA methylation changes in association with adverse prenatal or perinatal exposures and early-life exposures may manifest as adverse health conditions over time,^9,10,60,61,62^ where prenatal and antenatal factors, such as maternal health and health behaviors (eg, body mass index, hypertension during pregnancy, smoking, and psychological distress), have also been suggested to modify the methylome.^63,64,65^ Our current observation showing an association between PedBE age difference and brain growth at TEA agrees with this model, where it is plausible that the antecedents to preterm birth may have contributed to the observed variance in accelerated aging that manifests at TEA, rather than preterm birth itself combined with neonatal infection and comorbidities, and that these persist to early childhood, as evident at 18 months’ follow-up. Future studies to investigate the extent to which these factors contribute to PedBE in very preterm neonates will help delineate these associations.

### Strengths and Limitations

Our study has some strengths, including our combining of epigenetics with neuroimaging modalities to understand neonatal brain growth and development. Our use of the PedBE clock, which is particularly tailored to the pediatric population and is emerging as an accurate tool for measuring biological aging among children from noninvasive buccal samples,^28,29^ is another strength of this work. In contrast to previous epigenetic clocks trained in the placenta or cord blood tissues, the use of buccal tissues allowed us to investigate DNA methylation alterations during the neonatal period at more than 1 time point. Using a prospective cohort of deeply phenotyped neonates with longitudinal follow-up is also a strength of this work. Because large contemporary cohorts show that almost one-fourth of extremely preterm neonates have sepsis,^66^ we suggest that our findings are relevant to understanding brain health in this clinical population.

Our study also has some limitations, including the relatively small sample size, which warrants further investigations using larger cohorts. In addition, because more than half of the sample had a comorbidity, such as brain injury, the results of this work may not be generalized to the entire population of very preterm neonates. Another limitation of our work is the lack of genotyping data and the incomplete data on self-reported racial and ethnic background of the participants. Although we were not able to account for all the genetic differences in this sample, we attempted to capture ancestry-specific associations by using population-specific DNA methylation patterns. DNA methylation signatures are tissue specific, which may have implications for the interpretability of the associations between brain findings and the epigenetic changes in the buccal epithelium. However, epigenetic epidemiological studies have suggested that samples from peripheral tissues, particularly the buccal epithelium, can be used as surrogate tissues for detecting DNA methylation changes occurring in the brain because of the strong correlation of DNA methylation patterns in both tissues, which in turn may be associated with their common embryologic ectodermal origin.^67^ The training and testing population of the PedBE clock did not comprise preterm infants. We are therefore unable to infer that the PedBE clock sites are unequivocally informative in this clinical population. However, our cohort represented a unique opportunity to test its applicability to distinct populations, including very preterm and extremely preterm infants. Neonatal clocks are an emerging field of study^68^; however, the dynamics of DNA methylation among preterm infants may vary from term-born infants. Because the selection of CpGs for epigenetic clocks is based on machine learning algorithms and biological programming, it is reasonable to infer that an epigenetic clock trained exclusively on preterm infants will be useful. Overall, we are only beginning to explore epigenetic clocks in the context of pediatric development and its use in unique populations, such as preterm neonates. However, we anticipate that our work will add to the accumulating evidence that demonstrates the utility of the PedBE clock in diverse pediatric settings.

## Conclusions

The findings of this cohort study characterize the key variables that the PedBE clock reports on for very preterm neonates, suggesting it as a potential tool to assess brain growth and development among this vulnerable population, independent of other risk factors and neonatal comorbidities. Our results further suggest that epigenetic aging among very preterm infants may be associated with adverse exposures encountered very early in life, such as the stress of preterm birth, that may manifest by TEA. Our work provides a step forward in integrative approaches that can be used to tailor precision health tools and interventions that aim to identify and target very preterm neonates at high risk of impaired neurodevelopment along the life course. Moving forward, neurodevelopmental assessments and multimodal neuroimaging, along the growth trajectory, can be helpful in assessing whether the PedBE age difference will be associated with brain functions and neurocognitive outcomes as these children continue to develop.

## Notes

Supplement.
eMethods.
**eFigure 1.** Proportions of Buccal Epithelial and Immune Cell Types in Buccal Swab Samples **eFigure 2.** A) Inverse Association, Assessed by Spearman Correlations, Between PedBE Age and Gestational Age in the Cohort of Very Preterm Born Neonates at Early in Life Timepoint (Scan 1) and at Term-Equivalent Age (Scan 2). B) Non-significant Sex-Specific Differences in the Correlation Between PedBE Age and Gestational Age. C) Positive Association, Assessed by Spearman Correlation Between PedBE Age and PMA at Early in Life Timepoint (Scan 1) and at Term-Equivalent Age (Scan 2)
eReferences.
