# Supplementary material for: Association of Pediatric Buccal Epigenetic Age Acceleration With Adverse Neonatal Brain Growth and Neurodevelopmental Outcomes Among Children Born Very Preterm With a Neonatal Infection
Source: JAMA Netw Open. 2022 Nov 2;5(11):e2239796. doi: 10.1001/jamanetworkopen.2022.39796 (PMC9631102; doi:10.1001/jamanetworkopen.2022.39796)
Supplement: Supplement. — eMethods. eFigure 1. Proportions of Buccal Epithelial and Immune Cell Types in Buccal Swab Samples eFigure 2. A) Inverse Association, Assessed by Spearman Correlations, Between PedBE Age and Gestational Age in the Cohort of Very Preterm Born Neonates at Early in Life Timepoint (Scan 1) and at Term-Equivalent Age (Scan 2). B) Non-significant Sex-Specific Differences in the Correlation Between PedBE Age and Gestational Age. C) Positive Association, Assessed by Spearman Correlation Between PedBE Age and PMA at Early in Life Timepoint (Scan 1) and at Term-Equivalent Age (Scan 2) eReferences. [file jamanetwopen-e2239796-s001.pdf]

Supplementary Online Content

Gomaa N, Konwar C, Gladish N, et al. Association of pediatric buccal epigenetic age acceleration with adverse neonatal brain growth and neurodevelopmental outcomes among children born very preterm with a neonatal infection. *JAMA Netw Open.* 2022;5(11):e2239796. doi:10.1001/jamanetworkopen.2022.39796

eMethods.
eFigure 1. Proportions of Buccal Epithelial and Immune Cell Types in Buccal Swab Samples
eFigure 2. A) Inverse Association, Assessed by Spearman Correlations, Between PedBE Age and Gestational Age in the Cohort of Very Preterm Born Neonates at Early in Life Timepoint (Scan 1) and at Term-Equivalent Age (Scan 2). B) Non-significant Sex-Specific Differences in the Correlation Between PedBE Age and Gestational Age. C) Positive Association, Assessed by Spearman Correlation Between PedBE Age and PMA at Early in Life Timepoint (Scan 1) and at Term-Equivalent Age (Scan 2)
eReferences.

This supplementary material has been provided by the authors to give readers additional information about their work.

© 2022 Gomaa N et al. *JAMA Network Open.*

eMethods

Neonatal comorbidities

We focused on neonatal co-morbidities with well-established associations with brain growth and neurodevelopmental outcomes, including severe retinopathy of prematurity (ROP) (≥ Stage III), moderate or severe brain injury (any severity of punctate white matter injury [1]or intra-ventricular hemorrhage ≥Grade II), patent ductus arteriosus (PDA) (defined based on the Canadian Neonatal Network criteria and the Children's Hospital Network and Database with echocardiography evidence of a PDA shunt), bronchopulmonary dysplasia (BPD), and the number of infections e.g., sepsis, clinical infection, and histological chorioamnionitis.

Magnetic Resonance Imaging (MRI)

Neonates were scanned without sedation in a neonatal MRI transport incubator (Sree Medical Systems, Cleveland, USA) on a Siemens (Erlangen, Germany) 3 T Tim Trio MRI scanner at the Hospital for Sick Children, Toronto, using a single-channel neonatal head coil (Sree Medical Systems, Cleveland, USA). Two MRI scans were acquired: the first in early life at a median post-menstrual age (PMA) of 32.9 weeks (interquartile range [IQR]: 31.9–34.7) and the second at TEA at PMA of 43.0 weeks (IQR: 41.0-46.0). Anatomical images were acquired using a T1-weighted FLASH sequence (repetition time [TR]: 36msec, echo time [TE]: 9.2 msec, field of view [FOV]: 192 × 88 mm, voxel size: 1 mm isotropic). Resting-state fMRI was acquired using an echo-planar imaging sequence (TE: 50 msec, TR: 3 s, FOV: 192 mm, matrix: 64 × 64, slice thickness: 3 mm, 32 slices, 100 volumes).

Neurodevelopmental outcomes at 18 months

Children and their families returned to the neonatal follow-up clinics at both hospitals at 18 months, corrected for gestational age. Neurodevelopmental abilities were assessed using the Bayley Scales of Infant Development, 3rd edition (Bayley-III) Cognitive, Language and Motor composite scores.[2] At this visit, children were also examined by a pediatric neurologist and assessed for cerebral palsy and associated changes on the neurological exam (e.g., spasticity, hypotonia).

DNA methylation measurement and preprocessing

Buccal epithelial swab samples were preserved at room temperature until shipped to the laboratory at the Department of Medical Genetics, University of British Columbia for processing. DNA methylation was measured using the Illumina Infinium Human MethylationEPIC BeadChip platform (850K Array) using standard procedures.[3] Extensive sample quality checks such as sex checks using the DNA methylation values of the XY probes, outlier checks using *lumi* and *watermelon* R packages, and intensity checks were performed to identify poorly performing samples. To account for background fluorescence and dye bias, normal-exponential out-of-band (noob) correction was performed, and noob corrected data was used to calculate the PedBE ages.

Cell type estimation

DNA methylation is cell type specific, and since there may be variability within buccal epithelial cell proportions, it is important to consider that a change in DNA methylation may to some extent represent an average change across all cell types in the samples or may signify a change in the composition of different cell types. Therefore, we computed buccal cell type proportions and blood immune cell types using HEpiDISH which is an iterative hierarchical version of the EpiDISH R package.[4]As expected, proportions of blood immune cell types were close to zero, although a significant variation in buccal epithelial cell proportions across the samples was observed (eFigure 1). We therefore adjusted for buccal epithelial cell proportions in our subsequent crude analyses.

PedBE clock description

PedBE age was calculated using the online script which was accessed through the publicly available repository, GitHub (https://github.com/kobor-lab/Public-Scripts/blob/master/PedBE.Md). The PedBE clock was calculated via 1,721 DNA methylation profiles of typically developing children across an age range of 0-20 years old, as previously described [5]. Elastic net penalized regression was utilized to select 94 CpG sites which were highly predictive of chronological age with a median absolute test error=0.35 years. PedBE age difference was then calculated as the difference between the epigenetic age and PMA at each timepoint (MRI 1 and MRI 2), thereby providing two PedBE age difference values for each neonate.

Ethnicity/ancestry assessment

© 2022 Gomaa N et al. *JAMA Network Open.*

Since genotyping data was not available for the cohort and while self-reported race/ethnicity may not always accurately represent the genetic ancestry of an individual, we compared three published methods based on DNA methylation data to capture some of the ancestry-related differences in our samples.[6-8] For each method, we ran a principal component analysis (PCA) on the ancestry specific CpGs identified by these approaches and found that the first two PCs from all the three methods were highly correlated explaining >90% of variance. While these methods were not specifically developed and tested in buccal samples, they were still able to capture the major genetic differences in this cohort. Overall, we ran our models with and without the ancestry PCs to confirm our findings were not driven by any ancestry differences in the study sample.

© 2022 Gomaa N et al. *JAMA Network Open.*

eFigure 1. Proportions of Buccal Epithelial and Immune Cell Types in Buccal Swab Samples

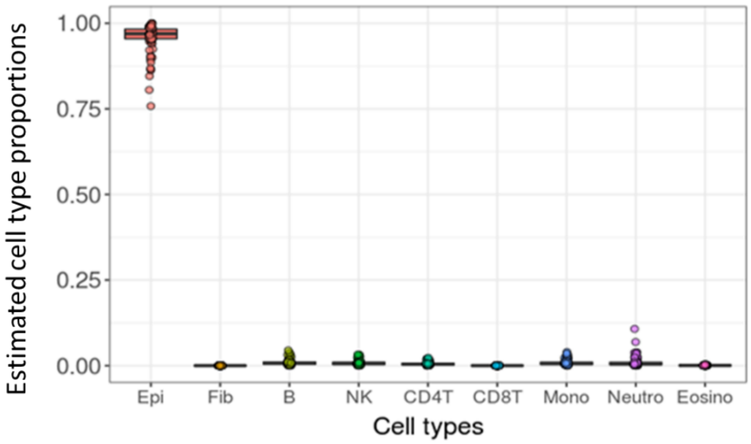

© 2022 Gomaa N et al. *JAMA Network Open.*

eFigure 2. A) Inverse Association, Assessed by Spearman Correlations, Between PedBE Age and Gestational Age in the Cohort of Very Preterm Born Neonates at Early in Life Timepoint (Scan 1) and at Term-Equivalent Age (Scan 2). B) Non-significant Sex-Specific Differences in the Correlation Between PedBE Age and Gestational Age. C) Positive Association, Assessed by Spearman Correlation Between PedBE Age and PMA at Early in Life Timepoint (Scan 1) and at Term-Equivalent Age (Scan 2).

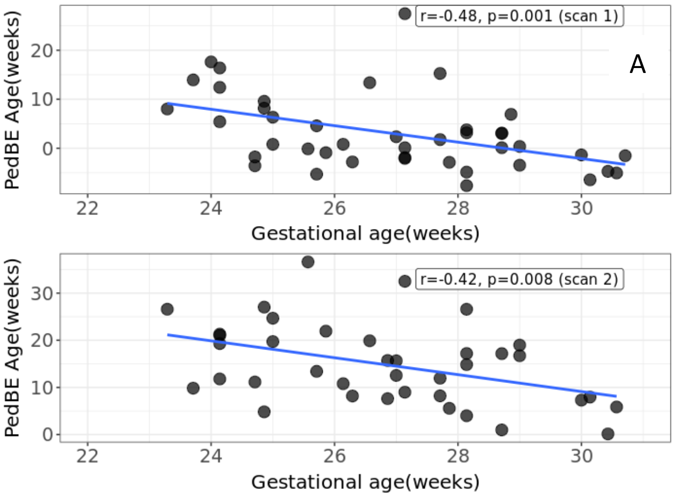

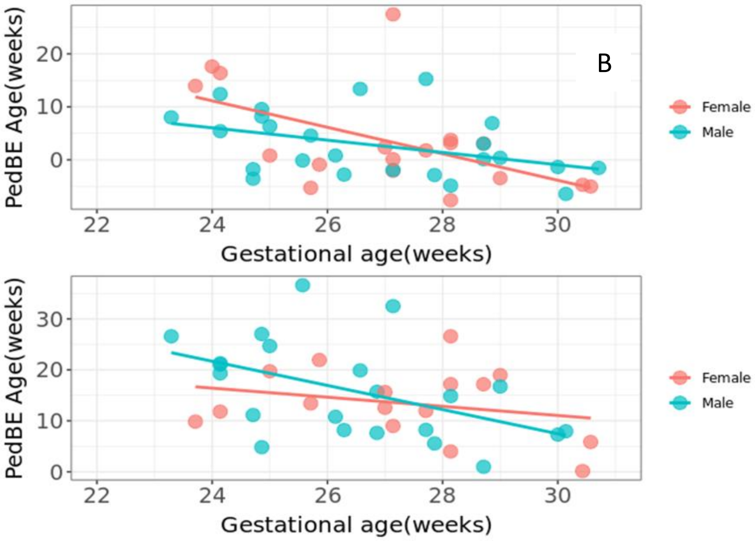

© 2022 Gomaa N et al. *JAMA Network Open.*

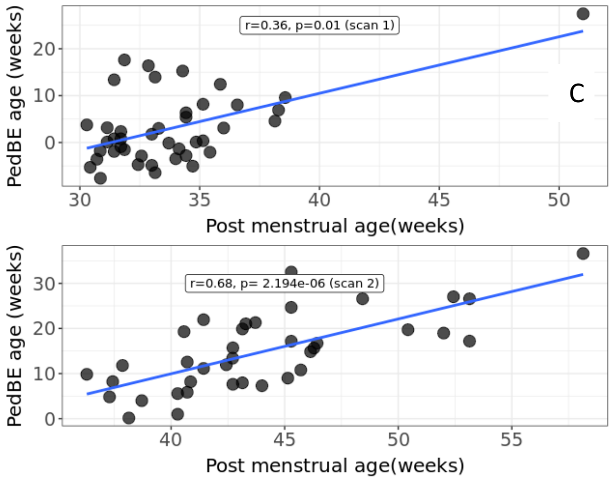

© 2022 Gomaa N et al. *JAMA Network Open.*

eReferences.

1. Guo T, Duerden EG, Adams E, et al. Quantitative assessment of white matter injury in preterm neonates. *Neurology*. 2017-02-14 2017;88(7):614-622. doi:10.1212/wnl.0000000000003606
2. Piñon M. Theoretical background and structure of the Bayley Scales of Infant and Toddler Development. *Bayley-III Clinical Use and Interpretation*. Elsevier; 2010:1-28.
3. Mcewen LM, Jones MJ, Lin DTS, et al. Systematic evaluation of DNA methylation age estimation with common preprocessing methods and the Infinium MethylationEPIC BeadChip array. *Clinical Epigenetics*. 2018-12-01 2018;10(1)doi:10.1186/s13148-018-0556-2
4. Zheng SC, Breeze CE, Beck S, et al. EpiDISH web server: Epigenetic Dissection of Intra-Sample-Heterogeneity with online GUI. *Bioinformatics*. 2019-11-09 2019;doi:10.1093/bioinformatics/btz833
5. McEwen LM, O'Donnell KJ, McGill MG, et al. The PedBE clock accurately estimates DNA methylation age in pediatric buccal cells. *Proc Natl Acad Sci U S A*. Sep 2020;117(38):23329-23335. doi:10.1073/pnas.1820843116
6. Barfield RT, Almli LM, Kilaru V, et al. Accounting for Population Stratification in DNA Methylation Studies. *Genetic Epidemiology*. 2014-04-01 2014;38(3):231-241. doi:10.1002/gepi.21789
7. Rahmani E, Shenhav L, Schweiger R, et al. Genome-wide methylation data mirror ancestry information. *Epigenetics & Chromatin*. 2017-12-01 2017;10(1)doi:10.1186/s13072-016-0108-y
8. Zhou W, Laird PW, Shen H. Comprehensive characterization, annotation and innovative use of Infinium DNA methylation BeadChip probes. *Nucleic Acids Research*. 2016-10-24 2016:gkw967. doi:10.1093/nar/gkw967

© 2022 Gomaa N et al. *JAMA Network Open.*
